# The Emulsification of Silicone Oil 5000 cs in Patients With Retinal Detachment

**DOI:** 10.7759/cureus.40205

**Published:** 2023-06-10

**Authors:** Hamza A Tayyab, Kashif Iqbal, Fawad U Rahman, Mashal Tayyab, Muhammad Awaid Abid, Uzair Asad

**Affiliations:** 1 Department of Ophthalmology, Layton Rahmatullah Benevolent Trust, Lahore, PAK; 2 Department of Ophthalmology, Mughal Eye Hospital, Lahore, PAK; 3 Department of General Surgery, Lahore General Hospital, Lahore, PAK

**Keywords:** emulsification, silicone oil removal, retinal detachment (rd), retinal detachment surgery, ophthalmology

## Abstract

Background

The emulsification of silicone oil is a significant concern for those with rhegmatogenous retinal detachment (RRD) following surgery. The aim of the study was to assess the frequency of emulsification in the patients who underwent primary vitrectomy and were administered 5000 cs silicone oil.

Methodology

The Layton Rahmatullah Benevolent Trust in Lahore conducted an ophthalmology study from January 2022 to March 2023. The patients who had primary vitrectomy for RRD with silicone oil tamponade were included regardless of age or gender. Those on anti-inflammatory or steroid medications prior to surgery were excluded. Retinal attachment was examined 8-12 weeks post operation to assess silicone oil removal eligibility. Emulsification occurrence was reported. Data on emulsification time, visual acuity (pre- and post-removal), mean intraocular pressure (IOP), and clinical outcomes were collected and analyzed using the Statistical Package for Social Sciences (SPSS) software (IBM SPSS Statistics, Armonk, NY). The results were graphically presented with mean, standard deviations, frequencies, and proportions.

Results

A total of 158 patients underwent silicone oil removal after undergoing primary vitrectomy for RRD with silicone oil. The mean age of the patients was 45.90 ± 17.8 years. The mean preoperative intraocular pressure (IOP) among the patients was 16.28 ± 2.97 mmHg. Post removal of silicone oil, the IOP was reduced to 12.66 ± 3.02 mmHg. In 11 out of 158 cases (6.9%) of RRD, emulsification had occurred with silicone oil 5000 cs. We found that out of 11 cases of emulsification, eight (72.73%) were 40 years or older in age. Seven (63.64%) patients had tamponade duration of 10 weeks or longer. However, the difference was not statistically significant.

Conclusion

In conclusion, our study found that the incidence of emulsification of 5000 cs silicone oil in the patients who underwent primary vitrectomy for the treatment of RRD was 6.9%. We observed that emulsification was more frequent in patients aged 40 years or older and those with a tamponade duration of 10 weeks or longer, although the difference was not statistically significant. Further investigation with bigger sample sizes and extended follow-up periods is required to verify our findings and explore potential factors that could lead to emulsification in this group of patients.

## Introduction

Retinal detachment is a serious eye condition that occurs when the retina, a light-sensitive layer of tissue in the back of the eye, is pulled away from its normal position at the back of the eye [1,2]. Silicone oil aids retinal reattachment in vitrectomy by pressing the retina to the eye wall, but its long-term effects can induce toxicity and cellular dysfunction. The necessity to remove silicone oil in a second surgical procedure also poses a certain risk of redetachment [2]. On the other hand, retinal detachment can also be attached by scleral buckling when there is no vitreous intervention, and the issue of silicone oil does not come at all. Silicone oil is favored in retinal detachment surgery due to its high viscosity and surface tension properties that offer superior visibility and structural support than other alternatives, facilitating more successful detachment repair. Its hydrophobic nature allows for long-term application and air travel, thereby improving overall recovery outcomes [2,3].

Silicone oil has been utilized increasingly in the correction of complicated retinal detachment cases, as it can help to prevent the formation of recurrent retinal detachment and support the healing process of the retina [3]. Additionally, silicone oil provides a longer tamponade effect than other agents, allowing for better retinal stabilization during the healing process. The use of silicone oil in retinal detachment surgery has become an important tool in the management of this condition and has significantly improved the visual outcomes of the patients [2-5].

Since 1962, silicone oils have been used in retinal detachment surgery. These methods have become more widespread, especially in challenging scenarios such as cases of extreme proliferative vitreoretinopathy, sizable retinal tears, diabetic retinopathy that spreads quickly, viral retinitis, and injuries to the eye [4]. The intraocular injection of silicone oil has significant clinical implications as it can lead to emulsification, resulting in a range of complications including obstructive secondary glaucoma, keratopathy caused by the disruption of corneal endothelium metabolic exchange, cataracts due to interference with lens metabolism, and retinopathy [4-7]. The rate of emulsification is largely determined by the physicochemical properties of the silicone oil [8].

One of the major drawbacks of using silicone oil is that it can sometimes become emulsified in the eye, which can lead to complications including glaucoma, cataracts, and inflammation [8,9]. Despite these potential drawbacks, there's a gap in the literature regarding the incidence, impacts, and risk factors associated with silicone oil 5000 cs emulsification in the patients undergoing retinal detachment repair. Thus, this study aimed to investigate the frequency of emulsification of silicone oil 5000 cs in the patients with retinal detachment and evaluate its potential effects on visual outcomes and complications. This research will deepen the understanding of silicone oil emulsification, aiding patient outcomes by refining surgical methods, improving follow-ups, and guiding the development of less emulsifying alternatives.

## Materials and methods

An observational prospective study was carried out at the ophthalmology department of Layton Rahmatullah Benevolent Trust between January 2022 and March 2023. The study was initiated subsequent to obtaining ethical approval from its institutional review board (IRB) under reference number GEN-LRBT/867. The enrollment of the patients followed a non-probability convenience sampling approach.

For sample size calculation, we adopted select statistical measures. Referring to a study by Ratanapakorn et al., we set the emulsification rate for the low-viscosity group at 63.64% and for the high-viscosity group at 40%. With a 95% confidence level and 80% power, we determined a sample size of 134 [9]. Individuals of all ages and genders who received primary vitrectomy with silicone oil 5000 cs endotamponade to address rhegmatogenous retinal detachment (RRD) were examined for potential inclusion. The patients who had consumed steroid or anti-inflammatory drugs for a minimum of three months prior to the surgical procedure were excluded from the study. Furthermore, individuals having inflammatory ocular conditions, such as uveitis, corneal scarring, or a past of scleral buckling, were also excluded from participating in this study.

All individuals underwent a comprehensive baseline assessment, including a detailed medical history, an ophthalmic examination, and imaging studies including optical coherence tomography (OCT), to establish a baseline of their ocular condition and function. We used the World Health Organization's (WHO) classification of vision impairment to categorize its severity. The WHO classifies vision impairment into two groups: distance and near-presenting vision impairment. The categories of distance vision impairment are as follows: i) mild, visual acuity worse than 6/12 to 6/18; ii) moderate, visual acuity worse than 6/18 to 6/60; iii) severe, visual acuity worse than 6/60 to 3/60; and iv) blindness, visual acuity worse than 3/60 [10]. Post-surgery follow-up was conducted at one week, one month, three months, and six months to monitor the occurrence of emulsification and any changes in ocular function. At each follow-up visit, all individuals were examined for complications such as intraocular pressure (IOP) elevation or cataract formation.

A thorough evaluation was conducted on all patients to assess their eligibility for the removal of silicone oil, with emphasis on the attachment of the retina within 8-12 weeks following the surgery. A single, impartial individual carried out the extraction process. To evaluate the emulsification of silicone oil, an extracted sample was compared against an unused sample of silicone oil 5000 cs procured from the same manufacturer. The samples were placed in an acrylic cuvette measuring 4.5 cm × 1.425 cm × 1.425 cm and having a volume of 1.5 cc to measure absorbance and transmittance changes using spectrophotometry (YR01852, Kalstein, Paris, France). Additionally, a macroscopic assessment was done based on the presence of a "fish egg" appearance on each silicon oil sample to determine the emulsification incidence in each case.

The researchers collected data on time intervals for emulsification, pre- and post-removal visual acuity, mean IOP, and other clinical outcomes. All data were analyzed using the Statistical Package for Social Sciences (SPSS) software version 23 (IBM SPSS Statistics, Armonk, NY). Data were presented in graphical form. For all continuous variables, mean and standard deviations were computed, while for all categorical variables, frequency and proportions were included. To find the impact of confounding factors such as age and gender, chi-square test was applied. A p-value of <0.05 was adopted as the cutoff for significance. All data were anonymized and coded to maintain the patients' confidentiality and privacy.

## Results

Silicone oil removal was performed in 158 patients who had previously undergone primary vitrectomy for rhegmatogenous retinal detachment (RRD) with silicone oil. The mean age of the patients was 45.90 ± 17.8 years. The mean preoperative intraocular pressure (IOP) among the patients was 16.28 ± 2.97 mmHg. Post removal of silicone oil, the IOP was reduced to 12.66 ± 3.02 mmHg. There was a predominance of males, and the majority had an involvement of the right eye. Furthermore, the majority of the patients had severe eye impairment (Table 1).

**Table 1:** Demographic and clinical characteristics of the patients with 5000 cs tamponade of silicone oil

Characteristics	Frequency (%)
Age category	
18-40 years	90 (56.96%)
40 years and older	68 (43.04%)
Gender	
Male	107 (67.72%)
Female	51 (32.28%)
Eye involved	
Right	96 (60.76%)
Left	62 (39.24%)
Impairment of vision	
Mild impairment	2 (1.27%)
Moderate impairment	21 (13.29%)
Severe impairment	135 (85.44%)
Time of tamponade (weeks)	
<10 weeks	71 (44.94%)
>10 weeks	87 (55.06%)

In 11 out of 158 cases (6.9%) of RRD, emulsification had occurred. The study did not find any association of patient characteristics with the occurrence of the emulsification of silicone oil. We found that out of 11 cases of emulsification, eight (72.73%) were 40 years or older in age. Seven (63.64%) patients had tamponade duration of 10 weeks or longer (Table 2).

**Table 2:** Association of patient characteristics with the occurrence of the emulsification of silicone oil NKCM: no known comorbidity

Patient Characteristics	Emulsification Positive (N = 11)	Emulsification Negative (N = 147)	P-Value
Gender			
Male	5 (45.45%)	102 (69.39%)	0.101
Female	6 (54.55%)	45 (30.61%)	
Age (years)			
18-40 years	3 (27.27%)	87 (59.18%)	0.039
40 years and older	8 (72.73%)	60 (40.82%)	
Comorbidities			
Hypertension	5 (45.45%)	102 (69.39%)	0.101
Diabetes	6 (54.55%)	45 (30.61%)	
NKCM			
Time of tamponade (weeks)			
<10 weeks	4 (36.36%)	67 (45.58%)	0.553
>10 weeks	7 (63.64%)	80 (54.42%)	

## Discussion

The extensive utilization of silicone oil in surgeries involving the retina and vitreous humor has generated significant apprehension regarding the potential risks and adverse effects linked to the temporary usage of silicone oil as a tamponade during postoperative care [9-13].

In a study by Ratanapakorn et al. [9], the objective was to compare the emulsification rate of silicone oil to various viscosities in the patients undergoing complicated retinal detachment surgery. The study enrolled 60 patients who received silicone oil tamponade for the surgery [9]. The findings of the study indicated no significant difference in the rate of emulsification between the three groups. The emulsification rate was assessed using ultrasound biomicroscopy at one, three, and six months after the operation. The study observed a rise in the emulsification rate in all three groups, with the highest rate at six months post-surgery. The effect of age, gender, and axial length on the emulsification rate was also evaluated in the study. The outcomes revealed that age and axial length were significantly associated with a higher emulsification rate, while gender did not have a significant effect [11].

The study by Toklu et al. aimed to investigate the time course of silicone oil emulsification following its use in vitreoretinal surgery [12]. The authors found that silicone oil emulsification was a gradual process that could take several months to develop. The degree of emulsification was observed to increase with time, reaching a peak at around 12 months post-surgery. Additionally, the study demonstrated that emulsification was more pronounced in the patients with higher levels of inflammation and those with a history of ocular surgery. The authors also noted that the emulsification process could lead to complications such as increased intraocular pressure and cataract formation [12].

Although most eyes demonstrate the expected time course for silicone oil emulsification, there is a specific condition that has been observed to hasten this process. Yilmaz and Güler's study showed that the patients with underlying nystagmus experience faster silicone oil emulsification than previous reports in the literature. They found that all eight eyes with nystagmus exhibited emulsification within one to three months after surgery. The authors suggest that the repeated shear force exerted on the silicone oil due to the ongoing nystagmus contributes to the acceleration of emulsification [13].

Miller et al. thoroughly evaluated the adverse outcomes that may arise from emulsified silicone oil post-retinal detachment surgery [14]. They emphasized that emulsification is a common complication that can lead to diverse issues, including elevated intraocular pressure, the development of cataracts, and harm to the corneal endothelium. The severity of these complications was found to be directly proportional to the extent of emulsification, with more severe cases requiring surgical intervention to remove the emulsified oil. In addition, the authors highlighted the importance of careful patient selection and informed consent, as well as the need for vigilant postoperative monitoring to detect any early signs of emulsification. They also discussed the potential use of newer, less emulsifiable silicone oil formulations as a way to minimize the risk of complications. Overall, the study provided a useful overview of the potential complications associated with emulsified silicone oil, emphasizing the need for careful management and monitoring in the patients undergoing retinal detachment repair [14].

Our study reported a reduction in IOP after the removal of silicone oil. Numerous researches claim that the length of silicone oil tamponade has no impact on the rise of IOP [15]. On the other hand, investigations have shown that prolonged tamponade is in fact a significant risk factor for vision loss [16]. The patients who received vitrectomy and silicone oil endotamponade treatment for rhegmatogenous retinal detachment without macular involvement have been found to experience severe vision loss. According to reports, the patients with good preoperative best-corrected visual acuity (BCVA) have experienced vision loss during silicone oil endotamponade or after silicone oil removal without any apparent cause [16]. In a study by Christensen and la Cour, the visual outcomes of the patients who underwent vitrectomy for macula-on retinal detachment with either silicone oil or gas endotamponade were evaluated. It was noted that silicone oil had a 30% rate of unexplained loss of vision [17]. The only factor associated with the reported vision loss that was statistically significant was the length of silicone oil tamponade [16]. Some studies claim that silicone oil emulsification is essential for IOP rise during retinal detachment surgery but has little bearing on how long silicone oil endotamponade lasts [18]. A rise in IOP has been associated with early postoperative silicone oil overfill, pupillary blocks, the flow of silicone oil into the anterior chamber, inflammation following surgery, and ocular hypertension following the use of steroids. Aphakia, iris neovascularization, preexisting glaucoma, and chronic uveitis are the key risk factors for early secondary glaucoma [19].

A study conducted by Vidne-Hay et al. included 41 patients and 43 eyes treated with silicone oil tamponade over a span of five years. The rates of complications, visual acuity, and retinal reattachment were the parameters assessed. Before the vitrectomy with silicone oil tamponade, 93% of the eyes had at least one ocular procedure done previously. The percentage of retinal attachment was 55.8%. It was found that 33.3% of the cases developed emulsification. Other complications included glaucoma formation in 28.6% of the cases, band keratopathy in 21.4% of the cases, and corneal decompensation in 16.7% of the patients [20].

Despite the fact that this study sheds important light on the prevalence of silicone oil 5000 cs emulsification in the patients with rhegmatogenous retinal detachment (RRD), a number of limitations should be noted. Firstly, this was a single-center study, and the participants were recruited using non-probability convenience sampling techniques; thus, the results could not be applicable to different demographics or therapeutic contexts. Secondly, the follow-up time was only six months. Therefore, to corroborate the study's findings and investigate potential risk factors for emulsification, longer follow-up and a multicenter study are required. To offer a more thorough understanding of the emulsification of silicone oil in patients with RRD, future studies should address these limitations.

## Conclusions

The results of the research showed that emulsification happened to 6.9% of the patients who got 5000 cs silicone oil after their initial vitrectomy for RRD treatment. It was observed that emulsification occurred more frequently among patients aged 40 years or above and those with a tamponade duration of 10 weeks or more, although this finding was not statistically significant. More detailed studies with larger sample sizes and extended follow-up periods are required to confirm these results and pinpoint possible risk factors for emulsification in this group of patients.
